# Social Interaction Needs and Entertainment Approaches to Pregnancy Well-Being in mHealth Technology Design for Low-Income Transmigrant Women: Qualitative Codesign Study

**DOI:** 10.2196/mhealth.7708

**Published:** 2018-04-13

**Authors:** Hana AlJaberi

**Affiliations:** ^1^ Purdue Polytechnic Institute Department of Computer Graphics Technology Purdue University West Lafayette, IN United States

**Keywords:** mHealth, mobile health, participatory design, pregnancy, Caribbean, immigrant women

## Abstract

**Background:**

Low-income Caribbean transmigrant women face unique health challenges during pregnancy that set forth multidimensional implications for the design of mobile health (mHealth). Acknowledgment of the unique health needs of low-income Caribbean immigrant women in the United States and what that entails regarding technology design remains rarely examined in the literature of mHealth technologies.

**Objective:**

The goal of this study was to reveal the needs and gaps in mHealth interventions for pregnant immigrant women not yet realized in this field. These understandings reveal design opportunities for mHealth.

**Methods:**

The use of the qualitative participatory action research approach of codesign workshops in this study resulted in design solutions by the participants after reflecting on their earlier focus group discussions. The highlights are not the resulting designs per se but rather the inferences derived from the researcher reflecting on these designs.

**Results:**

The designs exposed two themes relevant to this paper. First, the participants desired the inclusion and rebuilding of social and organizational relationships in mHealth. The resulting designs formulate an understanding of the women’s health-related social support needs and how technology can facilitate them. Second, the participants wanted entertainment with an element of social participation incorporated in mHealth pregnancy management interventions. This brings attention to the role entertainment can add to the impact mHealth can deliver for pregnancy well-being.

**Conclusions:**

The study concluded with an examination of social and entertainment design implications that reveal pregnant immigrant women’s virtual health-related sharing habits, choice of sharing interaction scenarios during pregnancy (eg, local, long distance, one-way, two-way, and many-many), and choice of sharing media (eg, text, voice, and video). Additionally, the study revealed exclusions to social sharing capabilities in health technologies for these women.

## Introduction

### Transnational Social Support

What constitutes social support is the feeling of one’s being cared for and assisted as part of a loving social circle [1]. Transnational social support within the context of immigrants is about accessing social support resources in the receiving country, while also maintaining existing ties in the origin country [2]. The term *transmigrants* was coined to describe immigrants that neither limit themselves to their geographical origin nor to the limits of the new migratory space [2]. Instead, they proactively and creatively partake in new ways in developing a new sense of self and maneuvering creative routes to resources to help the new self. The conception of the term transnational social support is accredited to technological advances in communication technologies such as the use of the Internet and cellular phone capabilities [2] and hence, the use of the term transmigrants at times throughout this research study to reference the participants.

There is literature advocating for Web-based health-related social sharing across borders [3,4], connecting people across different parts of the world. Additionally, there is other literature advocating for virtual mobilization of local communities with shared issues [5,6] in a term referred to as “community computing” [7].

### Mobile Health and Immigrant Women

Prenatal health is especially critical for low-income recent immigrants who face many health-related challenges as they adjust to their new host country. Their health is compromised as it is because of the unique challenges with this minority group of not knowing the medical resources available to them in the host country, cultural insensitivity by doctors and nurses [5], and mental health stigmas [8]. Thus, pregnancy only adds to their health vulnerability. Pregnancy is an ideal phase for intervention to achieve lifelong health changes, as many are unaware of the benefits associated with preventative care. During this critical time, women are open-minded toward health information and are more likely to follow through [9-11].

The literature on mobile health (mHealth) technologies is saturated with studies investigating contexts in developing countries. As such, the World Health Organization encourages efforts to be extended to the ignored context of minorities in developed countries [12].

### Prosocial Health Technologies

Several technologies promote health initiatives through social motivation or social pressure [3,13-16]. An example included applications that publish the user’s physical activity performances to their social media profile for others to see. This exposure motivates users through online encouragement from others or fear of being portrayed as an underachiever. Other forms of virtual support include online health communities, which provide a venue for social sharing, support, and health empowerment [6]. In addressing poor mental health in victims of domestic abuse among immigrant women in the United Kingdom, Clarke et al [17] introduced the digital technology means of photo sharing and storytelling to promote coping and mental wellness through peer support. Another example was a mobile phone app facilitating social sharing of knowledge about healthy eating from personal experiences in low-income African American communities [6]. Engaging in online communities can help users feel empowered with information so that they are better prepared to make better health decisions [3].

### Aims of This Study

Due to the small number of studies exploring health-related technology tools for immigrant women in developed countries, this study aims to contribute in filling this gap. In addition, this paper pushes to the surface the consideration of issues, including emotional and social care to shed light on a new perspective for the typical topics of diet, activity, and weight tracking in pregnancy management. Thus, social health technology research is extended through the examination of mHealth as a social technology empowering pregnant transmigrant women with safe user-generated health-promoting content.

## Methods

### A Qualitative Study

The methods used in this study were approved by Purdue University Institutional Review Board. This qualitative study adopts a participatory framework within a critical theory paradigm [18], which underpins the choice of methods used. This paradigm helps to produce data informed by two processes: analyzing the interpretation of data and then suggesting an action agenda as recommendations for reform. The participatory framework provides the researcher with the opportunity to seek immediate and valuable input from stakeholders in the design of mHealth technologies to contribute strategic design decisions with the barriers they face daily in mind [19]. The following sections cover recruitment and sampling strategies, study procedures, and data analysis methods.

### Recruitment

The study participants were recruited over the course of 6 months (April 2015-September 2015) by email and in-person recruitment and through personal connections. Information about the purpose of the study along with criteria for eligibility were distributed by email through Healthy Mothers Healthy Babies organization in West Palm Beach, Florida. Additionally, flyers were displayed in public advertisement boards at grocery stores and college campuses around South Florida. In addition, the information was pitched through face-to-face contact with potential participants in public areas. The majority of participants were enrolled in this study by snowball sampling [20], as in word of mouth through personal connections of the author.

### Sampling

The target participants were determined using criterion-sampling strategy [20]. The purpose of employing criterion-sampling strategy was to include cases that showcase predetermined criteria that exhibit the potential to be “information rich” to uncover strengths and weaknesses that can be considered “targets of opportunity” for quality improvement of programs, systems, products, and so on. To be eligible, each participant must satisfy the following criteria: (1) be a Caribbean immigrant women living in South Florida, (2) low income with an hourly minimum wage paid job, (3) had given birth to at least one child in the United States in the age range of 18 to 30 years and in the last 5 years. All other age groups were excluded because of higher risk birth complications that were outside the scope of this study, (4) be able to communicate in English, and (5) have basic knowledge of using cell phones and the Internet. However, the researcher did not directly collect characteristics information specific to each participant in order not to alienate the female participants and to make them feel protected. The study enrolled 12 participants divided into three sessions with 4 participants each.

### Procedures

The study used focus group discussions and participatory codesign workshops. The first phase of focus group discussions took 30 min to complete. Focus groups were used as a warm up for engagement in the second phase of codesign workshops. Participants were asked to discuss their prenatal experiences as recent immigrants related to topics of pregnancy, relationships, and technology. The second phase of codesign workshops took 50 min to complete **.** Codesign is a collaborative activity between the researcher and the participants, with the participants taking on the expert role in ideation and conceptualization of design ideas reflecting their personal experiences [21]. The participants were divided into two groups with 2 participants each to ensure equal participation. Each group was asked to come up with design solutions reflecting themes discussed in phase 1. Then, the groups were asked to exchange designs, critique them, and revise them if they were so inclined. The researcher assured participants that no design is considered right or wrong. They were reminded that this activity is not to test their sketching skills. So, the researcher demonstrated plain and simple examples of how to sketch ideas [5]. This demonstrated what the expectations of their sketching skills were to be to encourage participation by those who might be intimated. A script of the procedures is provided in Multimedia Appendix 1.

All sessions were audiorecorded, and lasted about 2 hours. One of the sessions took place in a conference room of a public library in West Palm Beach, Florida. The two remaining sessions took place on a quiet beach in Surfside, Florida. This is an unpopulated beach for most days of the week. These locations were chosen because of the proximity to where some of the participants worked.

### Data Analysis

Both focus group and codesign workshops were transcribed following each session. The researcher supplemented each transcription with notes on preliminary reflection and a set of possible codes attached to pieces of text. An inductive constant comparative method was used to review and compare transcripts to come up with codes using an iterative coding process. The codes emerged from the transcripts and were not assumed beforehand. The process was iterated several times until connections were established among low-level themes and later combined into broader themes. Only after codes have emerged, the researcher consulted with past literature to make sense of the data. In the following sections, excerpts from the transcriptions are referenced using the session number 1, 2, or 3, with either group 1 or 2 within that session, and then with either 1, 2, 3, or 4 for each participant in that session. For example, [1.2.4] refers to session 1, with group 2 of the codesign segment, and participant 4.

## Results

### Overview

**Figure 1:**
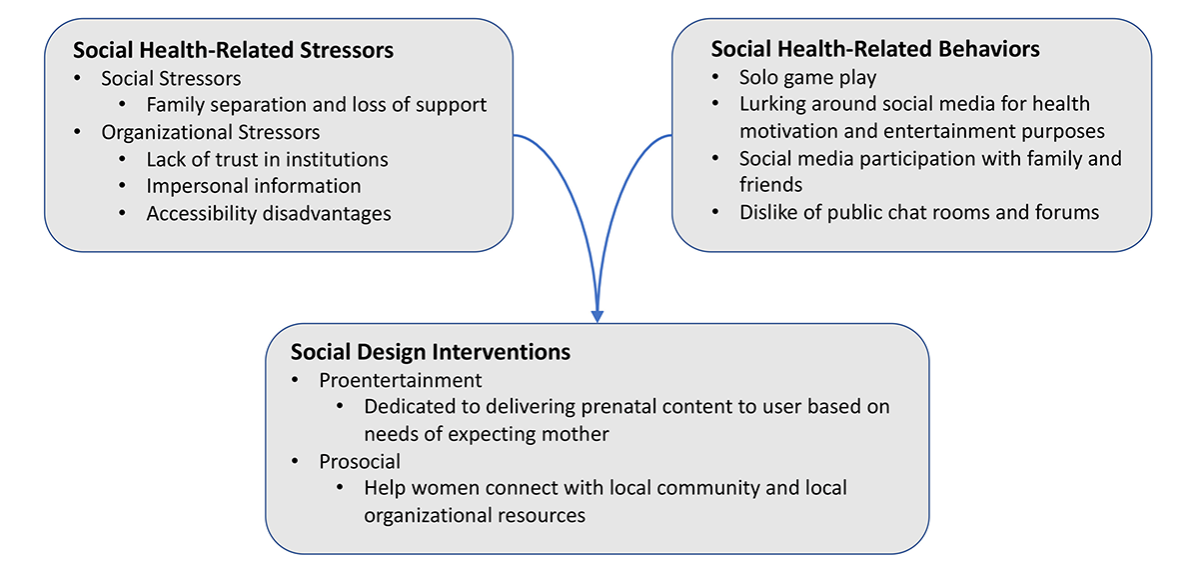
Themes related to social health-related stressors, social health-related behaviors, and social design interventions.

The results in the following section outline themes (Figure 1) related to social health-related stressors, social health-related behaviors, and social design interventions (Table 1) relevant to the context of transmigrant women.

### Social Health-Related Stressors

This section presents a brief summary of the social and organizational health-related stressors faced by pregnant immigrant women.

#### Social Stressors

For these Caribbean women, pregnancy is a celebrated occasion among female members of the family, such as with the mother and siblings, as illustrated in the following quote:

...men back home not his business you are pregnant. Bring your mama over to help you.

3.1.3

**Table 1:** Social design interventions relevant to transmigrant women.

Intervention		Description
**Proentertainment^a^**		
	Unlock My Pregnancy	Customized personal pregnancy lifestyle app
	Virtual Clinic	Prenatal clinic services “You Really Should” “All-Access” “Relaxation Suite”
**Prosocial^b^**		
	Buddy Network	Local caregiving social support system Reciprocal local support
	Video Call	One-to-one support Video, text, call, local doctors, or health care professionals Speak same language Understand the patient’s culture
	Conference Call	Three-way with patient, doctor, and family

^a^Dedicated to delivering prenatal content to user based on needs of expecting mother.

^b^Help women connect with local community and local organizational resources.

During this time, they help alleviate a lot of the burdens to allow the expecting mother to pursue wellness activities. Due to family separation and the loss of support such relationships play during pregnancy, many felt homesick, as illustrated in the following quote:

You are homesick when pregnant, or sometimes wish you have family or sisters or friends with you in appointments or when you need to make decisions or need the emotional support you know.

1.2.4

#### Organizational Stressors

Another relationship stressor for these women is at the organizational level. Their relationship with health care professionals were strained by mistrust, as illustrated in the following quote:

You coming here, you want to be part of the modern life. Is a hustle mama. They tell you all these things you need that you don’t need, or something wrong with you to charge you for tests you don’t need.

2.1.2

Their interactions and experiences were not pleasant, as illustrated in the following quote:

I ask the nurse at the clinic and she turn her nose up at me. The doctor dun speak in a language I understand then push me out.

3.1.1

Furthermore, it did not help that they used outdated, crammed, and impersonal informational mediums such as brochures and pamphlets, as illustrated in the following quote:

By the time I get home, you know, I already remove the brochure from my mind. By the time I get home, it is part of the trash if I remember that is somewhere. Mostly all wrinkled in my handbag.

1.2.1

Even when trust existed toward local organizations with resources catered specifically toward this population, the women could not make use of them because of fatigue and accessibility disadvantages, as illustrated in the following quote:

You have some organizations that give free access to pregnant women to professionals like social workers or free yoga classes and pregnancy lessons for, which is great. But, you have issues of commuting time and money to get there, when you already have to commute sometimes up to one hour or more on a bus everyday, sometimes for a job or two. Or maybe you have no time with job and family.

1.2.4

### Social Health-Related Behaviors

Variable energy and fatigue during pregnancy led some participants to feel isolated and bored. Equally, the changing pregnant body led some participants to be antisocial during pregnancy. In response, some participants found comfort in using gaming entertainment technologies, as illustrated in the following quote:

I play candy crush, is good when you stressed. It help your brain work again.

2.1.4

Additionally, some found lurking around social media sites as an entertainment strategy, as illustrated in the following quote:

I be addicted to finding out what’s goin on! I love lookin at the pictures and tweets while I’m sittin at home fat and lazy!

3.1.3

Surprisingly, few participants found lurking around social media to be a motivating strategy to engage in better prenatal health and wellness behaviors, as illustrated in the following quote:

I also sometimes go online and look for pregnant women pictures like Instagram and Pinterest, who dress up and workout, so I can be motivated.

1.1.2

Furthermore, some found comfort in connecting with family and friends by posting “pregnant selfies” to their mobile group messages or social media, as illustrated in the following quote:

I think I look better in pictures. You can use filters and if you are smart with how you pose, you will look sexy. You have more curves...So, I have a chance to celebrate my pregnancy. I can also say that I like when people like my pictures or put a comment things that I am glowing or say other nice things and it makes my day better because then I feel better about myself.

1.1.2

However, participants disliked sharing with strangers in chat rooms and forum features, as illustrated in the following quote:

If you have a good question, no one answer, no one care. Only if you a drama queen question, like my baby daddy drama, I don’t know what...Women are drama. They judge each other and rude to each other, mean, very mean.

2.1.1

### Social Design Interventions

#### Proentertainment Designs

Two design solutions in particular were dedicated to delivering prenatal content to the user based on the needs of the expecting mother. One is called *Unlock my Pregnancy* and another *Virtual Clinic.* Participants wanted to see content regarding prenatal diet and exercise, misconceptions, body changes, and emotional coping strategies. Participants described *Unlock my Pregnancy* as a customized personal pregnancy lifestyle app. On the other hand, *Virtual Clinic* is exactly as the name implies, a clinic. It was the participants interpretation of what prenatal clinic services should be about, all brought together virtually. The clinic is divided into three suites: *You Really Should, All-Access*, and the *Relaxation Suite*. The *You Really Should* suite is a lifestyle suite about healthy pregnancy diet and fitness. *All-Access* is a health suite with week by week content in relevant medical information. The *Relaxation Suite* within the *Virtual Clinic* endorses a range of relaxation strategies that participants referred to as “me time” (1.2.2), such as providing a weekly glossary on mood boosting foods, herbs, scents, beauty, and hair routines and added entertainment music, videos, and games. Additionally, the suite was equipped with support features from people in the immediate social circle and professionals as well. The goal for involving the immediate social circle was to boost the user’s mood through socializing and better communication. For example, the user may choose to display updates about their pregnancy mood by choosing from a list of mood emojis or sarcastic memes. Then, it sends push notifications to their social circle prompting them to react. They may react by initiating a Skype session, or an invitation for a joint activity, or vote from a set of system-generated relaxation tips, or send in their own recommendations. One of the participants described the value of these features by adding the following:

A lot of women feel lonely when pregnant, you are away from family and you bored because you wonder you can’t do the same things with friends like go dancing or go somewhere looking cute but you not. But, you don’t know how to communicate that. I think, ok, what I would do if I was back home now? You are always having family get together eat and talk hours and hours. Then I should try to do the same here because it’s fun and being social make you feel better. So now this maybe can help you communicate maybe that better with your husband, your family and friends back home, and your new friends here.

1.2.2

Furthermore, participants suggested adding a feature where you can chat with or send questions to a medical professional or therapist within relevant cultural organizations about emotional stressors. A user can view bios of these volunteers and therefore, ease their stress regarding with whom they communicate with. Additionally, it allows the user to ask questions without having to deal with the stress of interacting with strangers in forums with too many opinions and bullying.

#### Prosocial Designs

Participants offered recommendations to help women connect with local community and local organizational resources as a means of alleviating burdens and allowing more time to pursue wellness. One resulting design, the participants named *Buddy Network*, showcased a local caregiving social support system. Ultimately, the participant’s goal was building a reciprocal local support system, through which a two-way give and take platform allows people in the same community to share services and resources such as transportation, childcare, fitness companion, and so on. Here is what one participant shared about the added value to such a design:

When you first come to this country, something like this is really good. Also, its hard when you are not with your family and need help. Even if you have a man, maybe you feel like a single mother. Its good if a group of women want to help each other walk or run, like exercise, and anything else. I don’t know what I would do when I first come here, you know, if I didn’t have connections like kind neighbors or kind people in the church. It makes a difference.

2.2.2

Participants were quick to clarify that this is not similar to applications such as *Craigslist* or *Meetup.* To them, it is safer and more intimate and is built on a system of accountability, as illustrated in the following quote:

But, to take, you have to give back. It doesn’t have to be back to the same person. But, you can’t take without giving, or you can take and then it count as credit, and you can’t redeem another favor until you gave something...you subscribe to a community you live in. Maybe through local organizations connecting you, you can choose your own circles. Some features like that...We are thinking this is organized by community leaders, organizations, churches. So, then this can help with safety, also now you don’t have to deal with online bullying.

2.2.3

Another resulting concept for social support, *Video Call*, is a one-to-one support app that allows you to video, voice, or text chat with local doctors or other health professionals such as psychologists and nutritionists. After a very brief profiling step, the user will be connected with a professional who can speak the same language and can understand their culture. Participants expressed a preference for building a trusting long-term relationship with their doctors, as illustrated in the following quote:

What if I like them? Can I choose the same each time I use this? I think it will be nice to have the same professional every time. I will be willing to wait for them to be available if now I have a comfortable relationship with them, I feel like they know me, they actually know me! Now I don’t have to be stressed.

3.2.1

Several resulting designs came with both local and long distance virtual social support capabilities prompting others to participate in the intervention to aid in supporting the user. For example, *Unlock My Pregnancy* allowed screen-sharing capabilities so that a parent or a spouse can view and contribute to their profile, as illustrated in the following quote:

Maybe you have contributing days like #familysundays or #husbandsmonday. You see how I sneak that in?

2.2.4

Those who you allow to contribute to your profile are encouraged to participate by providing a “thumb up” if they like any content or “thumb down” if they dislike any content. One participant stated the following:

So you know what the people who care about you think, and you don’t have to ask them about every single thing when there is time difference or we busy. You know I would be curious, I would feel better too because I have companions with my decisions.

2.2.4

Similar to *Video Call*, *Unlock My Pregnancy* and *Virtual Clinic* also added social support features, enabling chat and question and answer sessions with volunteer local doctors, nutritionists, fitness instructors, and so on.

Another design, named *Conference Call*, is about having family accompany you virtually to all your prenatal activities so that you don’t have to feel homesick or alone. The concept allows you to share precious moments during pregnancy with family and friends no matter where everyone is. One participant stated the following:

You are homesick when pregnant, or sometimes wish you have family or sisters or friends with you in appointments or when you need to make decisions or need the emotional support you know. Even if they are in the same country or even city, sometimes you can’t both be there at same time. Or, you are even busy. We were talking about beautiful memories like skyping with sisters or friends showing how we prepare for a new child.

1.2.4

Participants acknowledged that it is similar to apps such as Skype or Facetime in a way, and they hope for it to be added as a feature to pregnancy apps. For example, to enable a three-way or more video call with in-person doctor appointments, or with a virtual specialist, or to stream prenatal classes, or to view videos together at the same time.

## Discussion

### Principal Findings

The desire for the inclusion of social relationships, rebuilding of organizational support, and incorporation of social entertainment in design make up the themes outlined in the findings of this study. The Discussion outlines design considerations of the role of transnational relationships, including the choice of sharing interaction styles and the role of entertainment in mHealth interventions.

### Consider the Role of Transnational Relationships

Due to the absence of family and the tensions in the relationships of the women with their significant other if he fails to adapt post migration into fulfilling these missing roles during pregnancy, the participants defaulted to transnational ties for social support during pregnancy. Several of the resulting designs further emphasized how transmigrant women value the important role social support plays in coping with pregnancy stressors. Thus, this study joins previous studies [5,22] that are prosocial design in health care interventions. However, within the context of this study’s transmigrant participants, the findings are rather protransnational social design in health care interventions. Incorporating the roles others play in a woman’s life is suggested in the literature for human-computer interaction (HCI) and health design interventions [5,22].

The transnational relationships in a transmigrant woman’s life fluctuate in influence and contribution power depending on her informational and emotional needs at a particular time throughout the course of pregnancy. Concluding from the participants’ accounts during focus group and design sessions, available prenatal technologies offer no social support capabilities despite the role relationships play in a pregnant transmigrant woman’s life. Social features and capabilities should enable valued transnational interactions to contribute in the women’s pregnancy mHealth interventions. Here is how the study envisions interaction scenarios that facilitate local, long distance, and individual caregiving themes the pregnant transmigrants showed interest within their resulting designs.

#### One-to-One Interactions

In this type of interaction scenario, mHealth allows others in the transmigrant pregnant woman’s intimate social circle to participate with aiding the pregnant woman in her health journey. Due to the significance in the meaning of family during pregnancy for immigrant women, inclusion of immediate family members such as the mother, siblings, and very close mom friends (moms with children of the same age) is a key design opportunity for mHealth. Another advantage to this type of interaction capability is the facilitation of an outlet to rebuild social relationships that play a major role in a woman’s pursuit of health behaviors, such as the role of the significant other.

Because the health care system and online sources fail them, the participants rely heavily on informal resources of information such as family and a close circle of mom friends. This could lead to socially and culturally influenced misconceptions that are common among pregnant women. This was evident during focus group discussions as women shared socially and culturally troubling discourses regarding fitness practices, food consumption, weight management, birthmark, fetal development, miscarriage causes, and more. The inclusion of these intimate relationships are beneficial in the sense that mHealth can play a role in controlling and filtering the type of information usually trickled down through such sources. This is one area where mHealth can make a significant impact.

Thus, this study calls for gender-neutral designs [22] so as not to discourage others from participation. Furthermore, consider adding social support enabling features such as interactive screen sharing, saved video chat messaging, the use of prompts, and creative contribution commands.

#### One-to-Many Interactions

A one-to-many interaction scenario provides a platform for rebuilding organizational support. The findings revealed that participants desire a caring connection with professionals whom they seek medical and wellness guidance from. This is consistent with findings in the literature regarding immigrant women’s health [5]. This was highlighted by designs such as *Conference Call, Video*, *Virtual Clinic*, and *Unlock my Pregnancy*. Additionally, participants desired such an interaction scenario in mHealth technologies that would help local community organizations provide their services virtually. The purpose is to allow for easy access by accommodating their busy lifestyle because of commuting time and fatigue. This type of interaction could allow the inclusion of one-to-one scenarios with transnational relationships to provide support and motivation to engage with organizational health activity resources.

#### Exclusions in Interaction Scenarios

Within previous HCI and social networking research [23-25], pregnant women are described as comfortable sharing pregnancy and motherhood information on online social settings, even with strangers. This certainly contradicts with the findings in this study and previous studies by Peyton et al [22] and Willcox et al [26]. Although the study by Peyton et al [22] does not explain this finding, Willcox et al [26] relate these findings to the women’s perceived risk of online bullying. What accounts for these contradictions are examined to a greater depth in this study. Let us examine what is been concluded from the findings on the participants’ social sharing habits during pregnancy and imposed impact of social sharing networks on the transmigrant’s pregnancy ecology.

In sharing with family, participants mostly preferred using private group texting apps such as Whatsapp and video chats such as Skype. Participants were comfortable sharing only in intimate social circles such as with parents, siblings, and few very close friends. The findings revealed that the women’s sharing habits in their personal social media profiles were conservative and cautious during pregnancy, with the exception of a few. Cultural beliefs, shaming, distrust over what people share on social media, and fatigue were some of the reasons for such conservative practices.

In the context of pregnancy, some transmigrants took on the role of viewers rather than sharers in interactions with strangers on social networking tools. Being a viewer allowed these participants to seek motivation and inspiration to engage in healthier activities. Others used it as an entertainment tool to feed curiosity and deal with boredom. Very few transmigrant participants did engage in online social media sharing during pregnancy in which their content was viewed by family members, coworkers, and acquaintances. There might be slight variations in sharing habits among transmigrants depending on factors such as age, exposure to pop culture, and the desire for a socializing support outlet while pregnant in a new country.

However, all participants seemed to have no desire for engaging with complete strangers online. All participants disliked chat rooms and forums in any Web or mobile pregnancy tool, which sometimes grouped strangers together who share the same birth month. They cited reasons of disapproval such as bullying, conflicting information, and responses that often go out on irrelevant tangents. As a result, participants did not feel comfortable engaging and sharing with strangers online within pregnancy tools.

#### Many-to-Many Interaction Scenarios

The only case of sharing with strangers the study’s participants felt at ease with were a community building form of social sharing. This theme was present in the resulting design *Buddy Network* that facilitated the concept of local caregiving. For immigrants, new ties in the host country contribute for an easy transition by providing help in guidance with navigating the new country, help with transportation, child care, and socializing [14]. What eases such interaction is that those who feel marginalized or excluded by the health care system and existing technologies come together because of shared circumstances to engage in a reciprocal relationship for the purpose of community building and organizational support. Such interventions can create opportunities for guided interaction within these groups to support and facilitate community sharing. The study suggests that such interventions be directed by local community organizations to ensure safety and accountability.

#### No Role Interactions

Not every design needs to include social support capabilities. Discussions during focus groups and one design by the study’s transmigrants, *Pregnancy Siri*, revealed that in certain instances participants preferred managing pregnancy independently and in others discretely. Therefore, designs that require no role from others are intended for the pregnant woman’s individual self-support use or individual private use. This conclusion is consistent with online information-seeking behaviors of Caribbean immigrant women [5]. In such cases, the built-in support capabilities of the technology itself are sufficient for achieving their needs. This presents an opportunity for future mHealth to examine in depth what entails designing for technologies that supports pursuing well-being discretely for transmigrant pregnant women. This certainly does not fit well with social networking technologies that publish to others [13,16], for example, the user’s fitness activities and progressions.

### Consider the Role of Entertainment

The female participants came up with designs to manage emotions and mental pregnancy stressors. They believed in the healing influence relaxation and entertainment activities provide in managing pregnancy health. Being healthy during pregnancy for these women goes beyond what has been predominately covered in prenatal health and HCI work that is been oriented toward topics such as dietary needs and weight management [9,22]. This is especially significant because of the emotional challenges reported by the participants and the likelihood of Caribbean women not receiving treatment for mental health issues [27] such as perinatal depression [28] and domestic abuse [17], which puts them at higher risk for prolonged mental distress than white American women.

Although this often ignored component was introduced in a previous study on immigrant women’s health by Brown et al [5], this study joins by endorsing the value of entertainment but in the context of pregnancy for transmigrants’ mHealth design. This means not only being proentertainment by advocating for relaxation activities as part of mHealth interventions [5] but also in adding a support aspect to it. This comes in a form where a technology prompts the important transnational ties in a pregnant woman’s life to contribute with recommended or joint entertainment activities.

### Limitations

The small sample size of participants may pose limitations with regard to the generalizability of the findings. The limitation of small sample size in this study was in part because of the extensive amount of time and work it took to engage and recruit these participants and schedule sessions with them. Additionally, the researcher was focused heavily on achieving depth to the emerging data and did not want to compromise the integrity of the data by focusing on a larger sample within the time constraints of this research. Small sample sizes are appropriate in certain research instances. Inductive exploratory research concerned with generating rich and multidimensional concepts from the data itself rather than being implied beforehand may benefit from small sample sizes [29,30]. In such cases, small samples can enhance the researcher’s role in recruitment and engagement with participants [29,30]. Furthermore, it allows for repeated access to participants, which strengthens the validity and reliability of the data [29,30].

### Conclusions

This paper focused on presenting two approaches to design imagined by the transmigrant participants of prenatal mHealth technologies. The first approach is for mHealth to facilitate the transnational social and organizational support and resources necessary for better pregnancy health management. The women formulating and then revising the resulting designs exposed attention to the role transnational relationships play in health and well-being and how technology can facilitate them. The second approach is employing entertainment and relaxation while accounting for social and cultural dimensions to bring pregnancy health into full circle for these women. Future work should examine transnationalism as a methodology for mHealth design to examine whether the country of origin for these immigrant women has the infrastructure to facilitate mHealth social interactions with the new host country. Finally, the value of transnational interactions in mHealth for potentially improving healthy behavior and technology use adoption may extend to other immigrant populations as well and prove beneficial beyond the period of pregnancy.

## Appendix

Multimedia Appendix 1 A script of the procedures used.

## Abbreviations

HCI: human-computer interaction
mHealth: mobile health
