# Supplementary material for: Social Interaction Needs and Entertainment Approaches to Pregnancy Well-Being in mHealth Technology Design for Low-Income Transmigrant Women: Qualitative Codesign Study
Source: JMIR Mhealth Uhealth. 2018 Apr 13;6(4):e61. doi: 10.2196/mhealth.7708 (PMC5924367; doi:10.2196/mhealth.7708)
Supplement: Multimedia Appendix 1 [file mhealth_v6i4e61_app1.pdf]

ppendix 1 Procedures Script

Focus Groups:

Reflect on your pregnancy experiences as an immigrant related to the following keywords (topics)?

Keyword	Moderator add-on probes
Pregnancy	• In your opinion, what does it take to have a healthy pregnancy?<br>• What was challenging during your pregnancy?<br>• How did you obtain your pregnancy information?
Relationships	• Whom do you most often communicate with in the US or your home country during your pregnancy?<br>• Can you describe the roles relationships with domestic partner, friends, and family play in your understandings of pregnancy?<br>• What are the strengths and weaknesses of each that might at times make one take precedence over the other?
Organizations	• Discuss the role of care providers and social or religious organizations whether in the US or your home country during pregnancy in the states?

Co-design Workshop:

Step 1: With your partner, in separate groups, come up with the worst design ideas you can possibly think of for technologies that offer solutions to pregnant immigrant women.

Step 2: Present it

Step 3: Now, design future interactive systems to turn those bad ideas to good ideas

Step 4: Each group present your ideas/sketches/designs

Step 5: Now, each group should swap designs. Then, each group take time to reiterate on the other group design. Think about the following when you iterate:

- What do you like or not like about it? What constraints or limitations you see to this design?
- How can you improve it? What can you add or remove from it to make it better?
- Which trimester are you designing for?
- Think of key themes like: individual use, one way use, two way use, physical,

emotional, information, local caregiving, long distance caregiving
